# Stochastic Thermodynamics of a Piezoelectric Energy Harvester Model

**DOI:** 10.3390/e23060677

**Published:** 2021-05-27

**Authors:** Luigi Costanzo, Alessandro Lo Schiavo, Alessandro Sarracino, Massimo Vitelli

**Affiliations:** Department of Engineering, University of Campania “Luigi Vanvitelli”, 81031 Aversa, Italy; luigi.costanzo@unicampania.it (L.C.); alessandro.loschiavo@unicampania.it (A.L.S.); massimo.vitelli@unicampania.it (M.V.)

**Keywords:** piezoelectric energy harvester, stochastic thermodynamics, work fluctuations

## Abstract

We experimentally study a piezoelectric energy harvester driven by broadband random vibrations. We show that a linear model, consisting of an underdamped Langevin equation for the dynamics of the tip mass, electromechanically coupled with a capacitor and a load resistor, can accurately describe the experimental data. In particular, the theoretical model allows us to define fluctuating currents and to study the stochastic thermodynamics of the system, with focus on the distribution of the extracted work over different time intervals. Our analytical and numerical analysis of the linear model is succesfully compared to the experiments.

## 1. Introduction

From microscopic organisms in the biosphere, life in general and human activities in particular critically depend on the conversion of different forms of energy into useful work. Harvesting energy from the environment is therefore a central task in many applications, where random fluctuations possibly arising from disparate sources at different scales, from the microscopic thermal Brownian motion in a fluid, to the macroscopic vibrations in means of transport, can be converted into work.

The well established rules of thermodynamics for macroscopic systems become blurred when fluctuations are relevant and have to be taken into account [1]. From a theoretical perspective, the study of fluctuations is addressed within the theory of stochastic thermodynamics, where the standard concepts of energy, heat, work and entropy, are extended to non-equilibrium systems, driven by external forces or coupled to different reservoirs. In this framework, the interest is focused on the fluctuations of the above quantities defined along a single trajectory in the stochastic motion of the system and on their probability distributions. Indeed, general relations have pushed the range of validity of standard thermodynamics into the realm of non-equilibrium regimes [2,3,4]: from the Fluctuation Relations [5,6,7,8,9] and the generalized fluctuation-dissipation relations [10,11], to the general results ruling work and heat exchanged in non-equilibrium transformations, such as the Jarzinski relation [12], the Crooks fluctuation theorem [13], or the Hatano–Sasa relation [14]. Very recently, thermodynamic uncertainty relations bounding the signal to noise ratio of a measured current have been also discovered [15]. In particular, the study of work and heat fluctuations has been the object of focus in several systems, such as overdamped linear Langevin Equation [16], particle diffusion in time-dependent potentials [17,18,19,20,21,22], Brownian particles driven by correlated forces [23], general thermal systems [24], asymmetric processes [25], underdamped Langevin Equation [26], or in transient relaxation dynamics [27]. The interest in these quantities is motivated by the search for optimization protocols in models of stochastic engines or, from a more theoretical perspective, by the general symmetry properties or by singular behaviors that work and heat distributions can show [28]. Experimental studies confirming theoretical predictions have been reported for instance in [29,30,31,32].

Energy harvesting model systems represent an interesting context where the concepts of stochastic thermodynamics can be applied, due to the fundamental relevance of random fluctuations. Very well studied examples are the Brownian (or molecular) motors [33], also known as ratchet models, where a probe is in contact with a thermal bath and the presence of a spatial asymmetry coupled to some non-equilibrium source allows to rectify the motion of the probe, with extraction of useful work. These systems have been studied theoretically and experimentally for instance in the context of granular media [34,35], where the dissipative interactions among grains induce the non-equilibrium behavior, or in biological motors [36], where active internal forces are at play.

**Figure 1:**
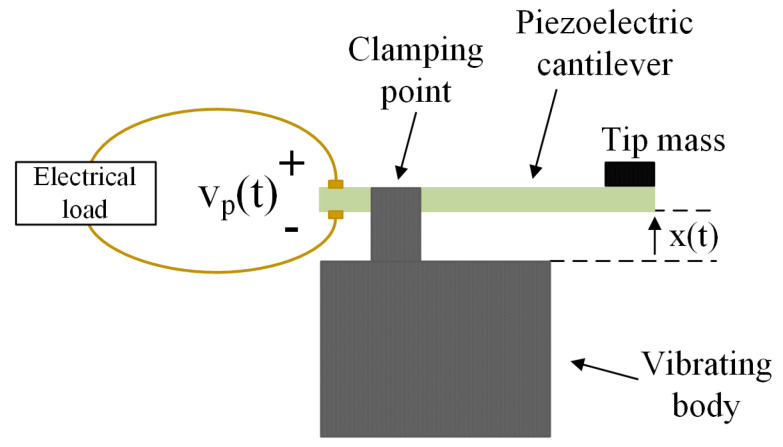
Schematic representation of a cantilever structure with piezoelectric harvester.

More application studies have been carried on in different kinds of energy harvesters, that are based on the piezoelectric properties of some materials. In this case, macroscopic vibrations of the system, as for instance in a car or in a train, can induce small currents, from which an output power can be extracted to feed sensors or small electrical devices [37]. Typically, piezoelectric harvesters are employed in resonant cantilever structures (see Figure 1). The mechanical to electrical energy conversion mechanism is based on the piezoelectric effect that is the ability of some materials (notably crystals and certain ceramics) to generate an electric voltage in response to an applied mechanical stress.

Due to their resonant nature, piezoelectric harvesters are typically studied in steady state sinusoidal conditions at frequencies belonging to their resonance band [38,39]. In particular, the main focus is on their energetic performance, that is on the mechanical and power electronic architectures and on the control techniques leading to the maximization of the extracted power [40,41]. Less attention has been devoted to the case of resonant piezoelectric harvesters excited by non-sinusoidal vibrations or solicited by white noise vibrations [42,43,44,45]. In particular, the theoretical analysis of [42] provided a stochastic description of the output power from resonant energy harvesters driven by broadband vibrations and output power dependence on signal bandwidth was considered. Instead, Ref. [44] proposed a methodology for the probabilistic analysis of a cantilever piezoelectric harvester under white Gaussian noise, without experimental validation. In [45], an experimental analysis on piezoelectric harvesters is carried out in the presence of harmonic, random and sine on random vibrations with particular reference to the electrical power extraction. However, in all previous studies on these energy harvesters no attention was devoted to the analysis of the fluctuations and distributions of relevant quantities such as the extracted power, in the general framework of stochastic thermodynamics of non-equilibrium systems.

Here we consider a typical piezoelectric harvester in a resonant cantilever structure driven by random broadband vibrations. Despite the several nonlinearities present in the experimental system, we show that a linear model with effective parameters can well reproduce the observed dynamics. In particular, we consider a mass in the presence of a harmonic potential, in contact with a source of white noise. The mass is also electromechanically coupled with a capacitor, which allow for power extraction through a load resistance. First, we show that the characteristic response curve, output power vs. load resistance, obtained from experiments is very well fitted by the analytical formula derived for the theoretical model. Then, we define a fluctuating work along a system trajectory, according to the standard approach of stochastic thermodynamics, and we focus on the study of the work fluctuations. We find that also the distributions of the work measured experimentally over different time intervals, are in very good agreement with those computed in numerical simulations of the linear model, using effective parameters.

Our study presents an experimental characterization of the distributions of integrated currents (output power) for a system that is used in applications as a valid energy harvester device. Moreover, our analysis shows that a simplified linear model, which allows for analytical computations, is able to accurately reproduce the experimental results, even at the fine level of fluctuations.

## 2. Experimental Setup

The schematic representation of the experimental setup for the piezoelectric harvester is shown in Figure 1. It consists of a cantilever structure with a tip mass, whose displacement in time x(t) due to the vibrations provided by the shaker, induces a voltage $v_{p}\left(t\right)$ across the electrical load.

**Figure 2:**
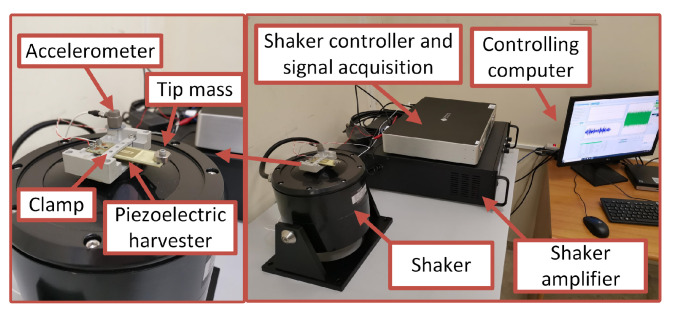
Picture of the experimental setup.

In all the experimental tests that we have carried out, we have used the commercial piezoelectric harvester MIDE PPA-4011 loaded by different electrical load resistances. This product incorporates four piezoelectric wafers resulting in significant performance improvements with respect to other models by MIDE. The harvester has been mounted on a shaker by using a support providing the possibility of different clamping positions. The addition of tip masses in order to define mechanical properties of the resonant structure is also possible. A picture of the whole experimental setup is shown in Figure 2. The shaker VT-500 by Sentek (500 N rated force and 450 m/s${}^{2}$ maximum acceleration) has been used to get the desired input vibrations. The controller Spider 81 allowed application of the desired voltage signal to the shaker amplifier and to carry out the recording, by means of its built-in acquisition board, of the voltage across the load resistance. An accelerometer 3055D2 by Dytran (sensitivity 100 mV/g on the range 50 g) has been used to monitor the applied acceleration in order to implement a closed-loop feedback vibration control.

**Figure 3:**
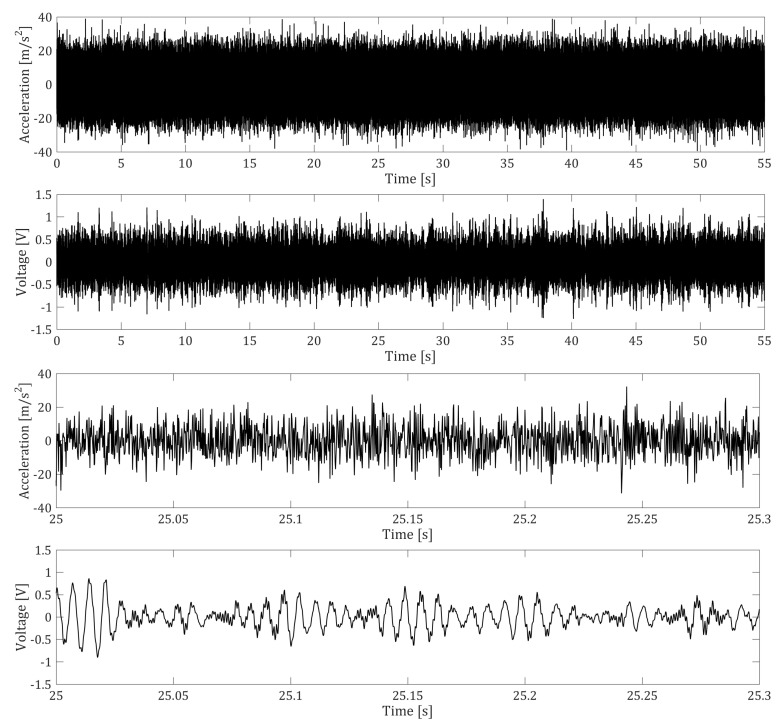
**Top**: Input white noise acceleration and voltage across a 2200 Ω load resistance. **Bottom**: zoom on a time window of 0.3 s.

In order to study the response of the system to broadband vibrations, we fed the shaker with a Gaussian signal generated with standard software (MATLAB), with a sampling rate f=5 kHz. In Figure 3 typical waveforms of the input acceleration and of the voltage across the load resistance (for R=2200Ω) recorded during experimental tests are shown.

**Figure 4:**
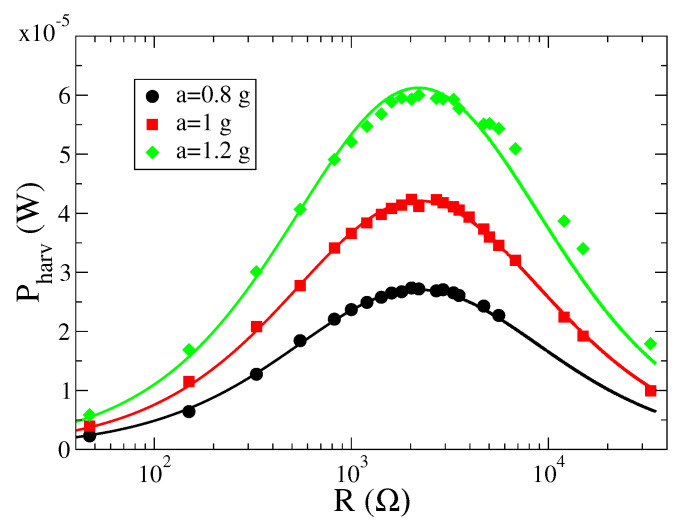
$P_{harv}$ (measured in Watt) as a function of the load resistance, for different values of the input acceleration *a*, measured in unit of the gravity acceleration *g*. Symbols are experimental data, while lines correspond to the formula (20).

The first quantity we analyzed is the extracted power $P_{harv}$ that can be obtained from the average dissipated heat on the load resistance for unit time, $P_{harv}=\langle v_{p}^{2}\rangle /R$. We show in Figure 4 the characteristic curves $P_{harv}$ vs. *R*, for different values of the input acceleration. From our analysis, we find an optimal resistance value $R^{*}∼2000\;\Omega$, which is independent of the shaking amplitude.

## 3. Theoretical Model

In order to describe the observed experimental results and to extend the study of the system to fluctuating quantities relevant in the stochastic thermodynamics framework, we consider the following linear model

(1) $$\begin{array}{ccc} \dot{x} & = & v \end{array}$$

(2) $$\begin{array}{ccc} M\dot{v} & = & -K_{s}x-\gamma vs.-\theta v_{p}+M\xi \end{array}$$

(3) $$\begin{array}{ccc} C_{p}{\dot{v}}_{p} & = & \theta vs.-\frac{v_{p}}{R}, \end{array}$$

where ξ is white noise with zero mean and correlation $\langle \xi \left(t\right)\xi \left(t^{\prime }\right)\rangle =2D_{0}\delta \left(t-t^{\prime }\right)$. In the above equations, *x* represents the displacement of the tip mass *M*, *v* its velocity, γ the viscous damping due to the air friction, $K_{s}$ the stiffness of the cantilever in the harmonic approximation, $v_{p}$ the voltage across the load resistance *R*, $C_{p}$ the effective capacitance in the circuit, and θ the effective electromechanical coupling factor of the transducer. In Equation (2) we have neglected the thermal fluctuations on the tip mass, which are too small to affect its motion.

In the system we can identify several characteristic times: $\tau_{1}=M/\gamma ,\tau_{2}=\sqrt{M/K_{s}},$ $\tau_{3}=C_{p}\gamma /\theta^{2},\tau_{4}=C_{p}R$. $\tau_{1}$ is the relaxation time of the tip mass due to the viscous damping, $\tau_{2}$ is the relaxation time within the harmonic potential, $\tau_{3}$ represents the typical timescale of the coupling between the proof mass and the capacitor, $\tau_{4}$ is the characteristic time of the RC circuit. Among the various characteristic times, $\tau_{3}$ is the only one depending on quantities belonging to both the electrical and the mechanical subdomain of the whole harvesting system. Hence, its physical meaning is not as intuitive as in the case of the other characteristic times. In any case, in order to better highlight its role, it is possible to show [41] that there is a link between the amplitude of the speed of the tip mass and the amplitude of the external acceleration, when the harvester operates in sinusoidal conditions at the resonance frequency and in open circuit (no load, R→∞). In particular, one has the relation [41]

(4) $$v_{max}=a_{max}\theta \frac{M}{\gamma \sqrt{1+\frac{1}{\left(K_{s}/M\right)\tau_{3}^{2}}}}=a_{max}\theta \frac{\tau_{1}}{\sqrt{1+\tau_{2}^{2}/\tau_{3}^{2}}}.$$

Therefore, the speed amplitude, in the above operating conditions, depends on all the three characteristic times, $\tau_{1}$, $\tau_{2}$, and $\tau_{3}$, that assume finite values. It does not depend on $\tau_{4}$, since it is unbounded in open circuit (R→∞).

The system of Equations (1)–(3) can be mapped onto a non-Markovian model, which makes clear how the presence of the coupling between the tip mass and the capacitor introduces a form of memory in the dynamics. In particular, one can rewrite the above equations as a generalized Langevin equation

(5) $$\dot{v}=-\int_{0}^{t}\left[2\frac{\gamma }{M}\delta \left(t^{\prime }\right)+\Gamma \left(t-t^{\prime }\right)\right]v\left(t^{\prime }\right)dt^{\prime }-\frac{K_{s}}{M}x+\xi ,$$

where the memory kernel Γ(t) has the simple exponential form

(6) $$\Gamma \left(t\right)=\frac{\theta^{2}}{C_{p}M}e^{-t/RC_{P}}=\frac{1}{\tau_{1}\tau_{3}}e^{-t/\tau_{4}}.$$

Let us note that here the friction memory kernel Γ(t) is not associated with any noise term. This puts the system by construction in a non-equilibrium state, because the fluctuation-dissipation relation of the second kind does not hold.

### 3.1. Average Values

The static properties of the linear model can be obtained by standard methods [46]. We introduce the column vector $X={\left(x,v,v_{p}\right)}^{T}$ and the coupling matrix

(7) $$A=\left(\begin{array}{ccc} 0 & -1 & 0\\ \frac{K_{s}}{M} & \frac{\gamma }{M} & \frac{\theta }{M}\\ 0 & -\frac{\theta }{C_{p}} & \frac{1}{C_{p}R} \end{array}\right),$$

so that the Equations (1)–(3) can be rewritten in vectorial form as

(8) $$\dot{X}=-AX+\eta ,$$

where $\eta ={\left(0,\xi ,0\right)}^{T}$. Defining the covariance matrix $\sigma =\langle X^{T}X\rangle$ as

(9) $$\sigma =\left(\begin{array}{ccc} \sigma_{xx} & \sigma_{xv} & \sigma_{xv_{p}}\\ \sigma_{vx} & \sigma_{vv} & \sigma_{vv_{p}}\\ \sigma_{v_{p}x} & \sigma_{v_{p}v} & \sigma_{v_{p}v_{p}} \end{array}\right),$$

at stationarity one has the constraint

(10) $$D=\frac{A\sigma +\sigma A^{T}}{2},$$

where *D* is the noise matrix

(11) $$D=\left(\begin{array}{ccc} 0 & 0 & 0\\ 0 & D_{0} & 0\\ 0 & 0 & 0 \end{array}\right).$$

From Equation (10) one gets the following relations for the covariance matrix elements

(12) $$\begin{array}{ccc} 0 & = & \langle xv\rangle \end{array}$$

(13) $$\begin{array}{ccc} 0 & = & \langle v^{2}\rangle -\frac{K_{s}}{M}\langle x^{2}\rangle -\frac{\theta }{M}\langle xv_{p}\rangle \end{array}$$

(14) $$\begin{array}{ccc} 0 & = & -\frac{\gamma }{M}\langle v^{2}\rangle -\frac{\theta }{M}\langle vv_{p}\rangle +D_{0} \end{array}$$

(15) $$\begin{array}{ccc} 0 & = & \frac{\theta }{C_{p}}\langle vv_{p}\rangle -\frac{1}{C_{p}R}\langle v_{p}^{2}\rangle \end{array}$$

(16) $$\begin{array}{ccc} 0 & = & -\left(\frac{\gamma }{M}+\frac{1}{C_{p}R}\right)\langle vv_{p}\rangle +\frac{\theta }{C_{p}}\langle v^{2}\rangle -\frac{K_{s}}{M}\langle xv_{p}\rangle -\frac{\theta }{M}\langle v_{p}^{2}\rangle \end{array}$$

(17) $$\begin{array}{ccc} 0 & = & \langle vv_{p}\rangle -\frac{1}{C_{p}R}\langle xv_{p}\rangle . \end{array}$$

The stationary distribution is a multivariate Gaussian

(18) $$P\left(x,v,v_{p}\right)∼\exp\left[-\frac{1}{2}\left(\sigma_{xx}^{-1}x^{2}+\sigma_{vv}^{-1}v^{2}+\sigma_{v_{p}v_{p}}^{-1}v_{p}^{2}+2\sigma_{xv}^{-1}xv+2\sigma_{xv_{p}}^{-1}xv_{p}+2\sigma_{vv_{p}}^{-1}vv_{p}\right)\right],$$

where $\sigma^{-1}$ denotes the inverse matrix of σ. The explicit expressions of the elements of σ are reported in Appendix A.

The average output power is the heat dissipated into the resistance per unit time

(19) $$P_{harv}=\langle {\dot{Q}}_{diss}\rangle =\frac{1}{R}\langle v_{p}^{2}\rangle$$

and its explicit expression as a function of the parameters is

(20) $$P_{harv}=\frac{D_{0}M^{2}R\theta^{2}}{M\left(\gamma +R\theta^{2}\right)+C_{p}R\gamma \left(C_{p}K_{s}R+\gamma +R\theta^{2}\right)}.$$

### 3.2. Fitting the Model to Experimental Data

The linear model described by the Equations (1)–(3) contains several physical parameters that are directly controlled in the experiments and others that can be fitted to match the measured data. In particular, the parameter $D_{0}$ that quantifies the amplitude of the white noise is related to the acceleration *a* provided by the shaker and to the sampling rate 1/Δt of the input signal, $D_{0}=a^{2}\Delta t/2$, where Δt=1/f=0.0002 s. Furthermore, the parameters $K_{s}$ and *M* are related to the characteristic frequency of the system, which for the experimental apparatus is $\sqrt{K_{s}/M}=2\pi \times 140$ Hz. Finally, the capacitor $C_{p}$ is estimated as $C_{p}$∼490 nF. The other parameters can be fitted to the experimental data through the analytical expression (20) as a function of the load resistance *R*. For the case *a* = 9.81 m/s${}^{2}$, we obtain the following values for the model parameters: M=0.0083±0.0002 Kg, θ=0.0195±0.002 N/V, γ=0.359±0.05 Kg/s. This set of parameters is used also for other values of the shaker accelerations used in the experiments, a=0.8×9.81 m/s${}^{2}$ and a=1.2×9.81 m/s${}^{2}$, providing a very good agreement between analytical predictions and experimental data, as shown in Figure 4.

### 3.3. Stochastic Energetics

The theoretical model allows us to study fluctuations and distributions of thermodynamic quantities defined at the level of the single trajectory. In particular, according to Sekimoto [47], we define the heat exchanged along a trajectory in a time interval τ with the surrounding medium as

(21) $$Q_{ex}\left(\tau \right)=-\int_{0}^{\tau }\gamma v{\left(t\right)}^{2}dt,$$

and the energy fed into the system from the external driving as the integral of the injected power $P_{inj}=M\xi \left(t\right)v\left(t\right)$

(22) $$E_{inj}\left(\tau \right)=M\int_{0}^{\tau }\xi \left(t\right)v\left(t\right)dt.$$

The product in the above equation is meant according to the Stratonovich prescription. Note that the heat in Equation (21) is released toward the medium. Using the Langevin Equation (2), we can rewrite these two terms as follows

(23) $$\begin{array}{ccc} Q_{ex}\left(\tau \right)+E_{inj}\left(\tau \right) & = & \int_{0}^{\tau }\left[-\gamma v{\left(t\right)}^{2}+Mv\left(t\right)\xi \left(t\right)\right]dt\\ \; & = & \int_{0}^{\tau }\left[-\gamma v{\left(t\right)}^{2}+v\left(t\right)\left(M\dot{v}+K_{s}x+\gamma vs.+\theta v_{p}\right)\right]dt\\ \; & = & \frac{1}{2}M\left[v{\left(\tau \right)}^{2}-v{\left(0\right)}^{2}\right]+\frac{1}{2}K_{s}\left[x{\left(\tau \right)}^{2}-x{\left(0\right)}^{2}\right]+\theta \int_{0}^{\tau }v\left(t\right)v_{p}\left(t\right)dt\\ \; & = & \Delta E+W, \end{array}$$

where

(24) $$\Delta E=\frac{1}{2}M\left[v{\left(\tau \right)}^{2}-v{\left(0\right)}^{2}\right]+\frac{1}{2}K_{s}\left[x{\left(\tau \right)}^{2}-x{\left(0\right)}^{2}\right]$$

is the mechanical energy variation and

(25) $$W\left(\tau \right)=\theta \int_{0}^{\tau }v\left(t\right)v_{p}\left(t\right)dt$$

can be interpreted as the work performed by the harvester. Equation (23) represents the first principle for stochastic thermodynamic quantities. The average output power in the stationary state is then

(26) $$P_{harv}=\langle \dot{W}\rangle =\theta \langle vv_{p}\rangle =-\gamma \langle v^{2}\rangle +M\langle v\xi \rangle ,$$

where $\langle v\xi \rangle =D_{0}$ and the last equality follows from Equation (23) using the stationary result 〈ΔE〉=0.

The output power can be also expressed as the dissipated heat in the load resistance in the time interval τ

(27) $$Q_{diss}\left(\tau \right)=\frac{1}{R}\int_{0}^{\tau }v_{p}{\left(t\right)}^{2}dt,$$

which, exploiting Equation (3), can be related to the fluctuating work *W* by

(28) $$W\left(\tau \right)=Q_{diss}\left(\tau \right)+\frac{C_{p}}{2}\left[v_{p}{\left(\tau \right)}^{2}-v_{p}{\left(0\right)}^{2}\right].$$

This shows that the difference between the fluctuations of *W* and $Q_{diss}$ is a term non-extensive in time. In our system, we have numerically checked that, for the studied time intervals, these border terms can be neglected. However, more generally, there are systems where such terms can be relevant for fluctuations, see for instance [17,48].

**Figure 5:**
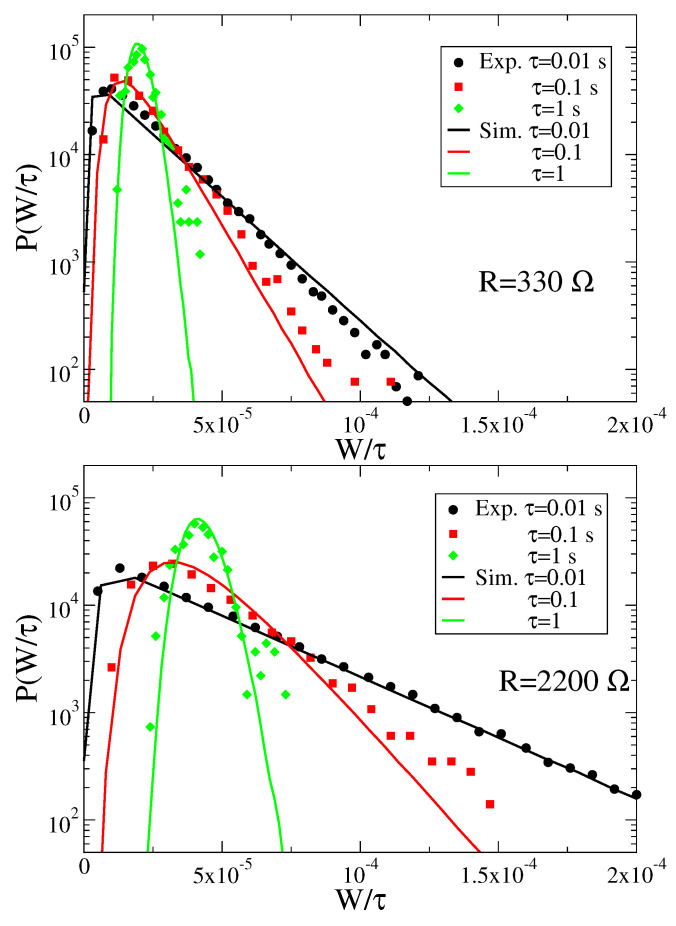

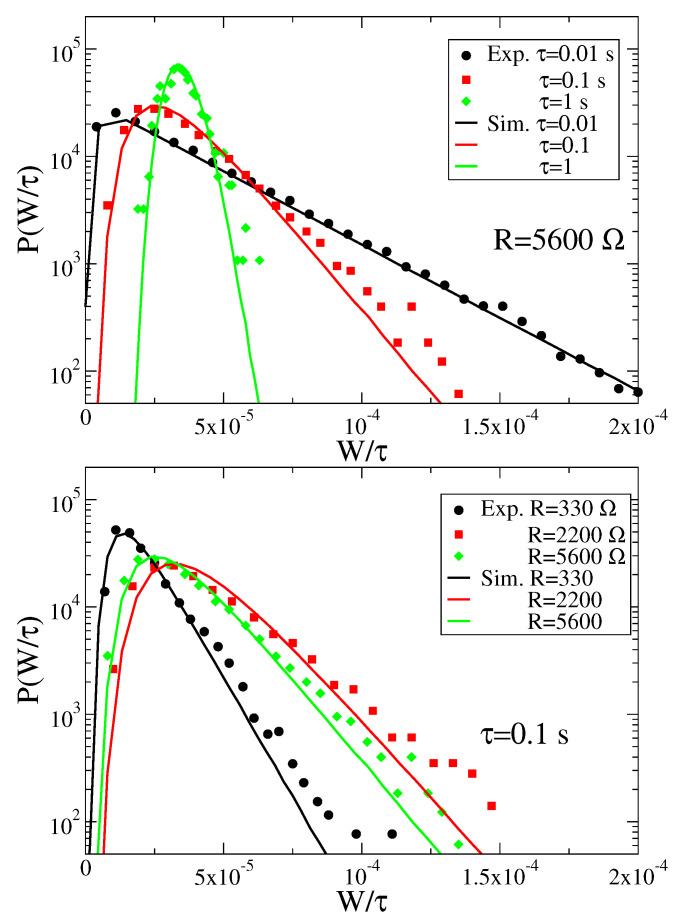
Distributions of W/τ (measured in Watt) for different values of the load resistance *R* and of the time τ. Dots represent experimental data and lines numerical results. Numerical simulations were obtained integrating the Langevin equation with a time step $dt=10^{-6}$, and averaging over ∼10${}^{5}$ realizations.

We have studied numerically and experimentally the work W(τ) for different values of τ. The experimental evaluation of this quantity has been obtained integrating the time series of the output signal for the voltage $v_{p}\left(t\right)$ (the whole recorded time series were 600 s long). The results are reported in Figure 5. First, we stress the good agreement between the work distributions obtained from experiments and numerical simulations. This shows that the linear model with white noise is able to accurately describe the experimental system, even at the level of fluctuations, in the range of explored parameters. The work distributions present a pronounced asymmetry for small time intervals τ, characterized by an exponential tail for large values of W/τ, as also observed for the functional form of the injected power distribution in the overdamped Langevin equation, obtained analytically in [16]. At large times, the distributions seem to converge towards a Gaussian form, symmetric around the mean.

**Figure 6:**
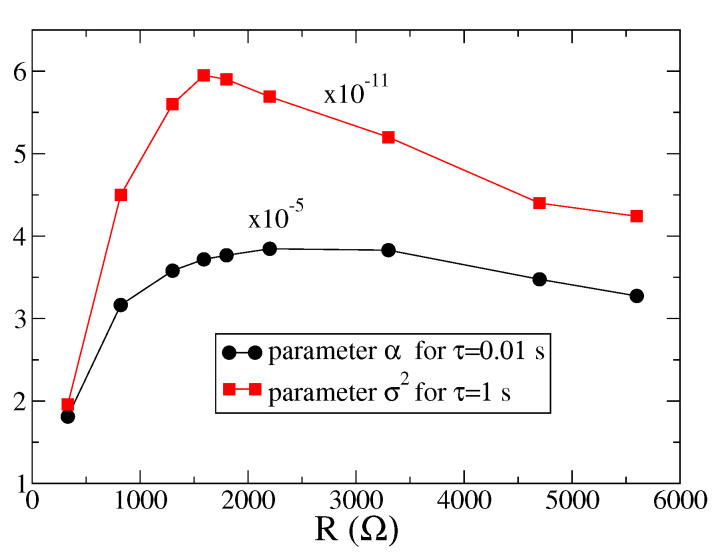
Parameters α and $\sigma^{2}$ obtained from the fit of the functions f(x) and g(x) to the experimental data, for different values of *R*.

In order to analyze the behavior of the work distributions measured in experiments as a function of the load resistance we have fitted the tail of the curves at short times (τ=0.01 s) with an exponential function f(x)∼exp(−x/α), extracting the parameter α. For the curves at large times (τ=1 s) we have fitted the data with a Gaussian function $g\left(x\right)∼\exp(-{\left(x-\mu \right)}^{2}/2\sigma^{2})$ to obtain the variance $\sigma^{2}$ as a function of *R*. The results are shown in Figure 6. We observe that both α and $\sigma^{2}$ have a non-monotonic behavior, with a maximum appearing around the value which maximizes the mean extracted power, showing that fluctuations are larger in proximity of the optimal working point.

## 4. Conclusions

We have studied experimentally a piezoelectric energy harvester driven by random broadband vibrations, focusing on the behavior of the extracted power as a function of the load resistance and of the vibration amplitude. We have shown that a linear model, consisting of an underdamped Langevin equation for the tip mass coupled with voltage dynamics reproduces very well the experimental data.

Moreover, the theoretical model allowed us to address the issue related to the behavior of the fluctuations of non-equilibrium currents, such as the extracted work in a time interval. This analysis plays a central role in the context of stochastic thermodynamics, where the properties of the system are scrutinized at the level of single trajectories. The comparison between the results of numerical simulations of the model and the experimental data showed that the linear system of equations provides a good approximation of the real system, reproducing the same behavior of the work distributions as a function of the parameters.

Our findings represent the first experimental study of the work fluctuations in a piezoelectric energy harvester and show that the observed behaviors can be consistently rationalized within a simple model, paving the way to future analyses. In particular, from the theoretical perspective, due to the linear nature of the model, the analytical computation of the work distribution and its large deviations function could be obtained with a path integral approach, as for instance described in [16]. Moreover, it could be interesting to modify the model by adding a noise source also in the equation for the voltage, obtaining a system of two coupled Langevin equations, or considering a bistable potential for the tip mass, as proposed in [49,50]. Analyses of other quantities such as heat or entropy production could be also carried out along the same lines. On the experimental side, it could be interesting to perform a similar study of current fluctuations in a system driven by realistic vibration sources, like cars or trains, taken from available databases, or where the simple linear resistance load is replaced by a diode bridge, as often considered in applications.

## Data Availability

Experimental and numerical data are available on request from the authors.

## Appendix A

Here we report the explicit expressions for the covariance matrix elements

(A1) $$\begin{array}{ccc} \sigma_{xx} & = & \frac{D_{0}M^{2}\left(C_{p}R\left(\gamma +C_{p}K_{s}R\right)+M\right)}{K_{s}\left(\gamma C_{p}^{2}K_{s}R^{2}+\left(\gamma +\theta^{2}R\right)\left(\gamma C_{p}R+M\right)\right)} \end{array}$$

(A2) $$\begin{array}{ccc} \sigma_{xv} & = & 0 \end{array}$$

(A3) $$\begin{array}{ccc} \sigma_{xv_{p}} & = & \frac{C_{p}D_{0}\theta M^{2}R^{2}}{\gamma C_{p}R\left(\gamma +C_{p}K_{s}R+\theta^{2}R\right)+M\left(\gamma +\theta^{2}R\right)} \end{array}$$

(A4) $$\begin{array}{ccc} \sigma_{vv} & = & \frac{D_{0}M\left(C_{p}R\left(\gamma +C_{p}K_{s}R+\theta^{2}R\right)+M\right)}{\gamma C_{p}R\left(\gamma +C_{p}K_{s}R+\theta^{2}R\right)+M\left(\gamma +\theta^{2}R\right)} \end{array}$$

(A5) $$\begin{array}{ccc} \sigma_{vv_{p}} & = & \frac{D_{0}\theta M^{2}R}{\gamma C_{p}R\left(\gamma +C_{p}K_{s}R+\theta^{2}R\right)+M\left(\gamma +\theta^{2}R\right)} \end{array}$$

(A6) $$\begin{array}{ccc} \sigma_{v_{p}v_{p}} & = & \frac{D_{0}\theta^{2}M^{2}R^{2}}{\gamma C_{p}R\left(\gamma +C_{p}K_{s}R+\theta^{2}R\right)+M\left(\gamma +\theta^{2}R\right)}. \end{array}$$

## References

[1] Puglisi A., Sarracino A., Vulpiani A. (2018). Thermodynamics and Statistical Mechanics of Small Systems. Entropy.

[2] Seifert U. (2012). Stochastic thermodynamics, fluctuation theorems and molecular machines. Rep. Prog. Phys..

[3] Marconi U.M.B., Puglisi A., Rondoni L., Vulpiani A. (2008). Fluctuation-dissipation: Response theory in statistical physics. Phys. Rep..

[4] Jepps O.G., Rondoni L. (2010). Deterministic thermostats, theories of nonequilibrium systems and parallels with the ergodic condition. J. Phys. A Math. Theor..

[5] Gallavotti G., Cohen E.G.D. (1995). Dynamical ensembles in stationary states. J. Stat. Phys..

[6] Evans D.J., Cohen E.G.D., Morriss G.P. (1993). Probability of second law violations in shearing steady flows. Phys. Rev. Lett..

[7] Searles D.J., Rondoni L., Evans D.J. (2007). The steady state fluctuation relation for the dissipation function. J. Stat. Phys..

[8] Rondoni L., Garbaczewski P., Olkiewicz R. (2002). Deterministic thermostats, fluctuation relations. Dynamics of Dissipation, Lecture Notes in Physics 597.

[9] Maes C. (1999). The fluctuation theorem as a Gibbs property. J. Stat. Phys..

[10] Baiesi M., Maes C. (2013). An update on the nonequilibrium linear response. New J. Phys..

[11] Puglisi A., Sarracino A., Vulpiani A. (2017). Temperature in and out of equilibrium: A review of concepts, tools and attempts. Phys. Rep..

[12] Jarzynski C. (1997). Nonequilibrium equality for free energy differences. Phys. Rev. Lett..

[13] Crooks G.E. (2000). Path ensemble averages in systems driven far from equilibrium. Phys. Rev. E.

[14] Hatano T., Sasa S. (2001). Steady-state thermodynamics of Langevin systems. Phys. Rev. Lett..

[15] Barato A.C., Seifert U. (2015). Thermodynamic Uncertainty Relation for Biomolecular Processes. Phys. Rev. Lett..

[16] Farago J. (2002). Injected Power Fluctuations in Langevin Equation. J. Stat. Phys..

[17] Baiesi M., Jacobs T., Maes C., Skantzos N.S. (2006). Fluctuation symmetries for work and heat. Phys. Rev. E.

[18] Kwon C., Noh J.D., Park H. (2011). Nonequilibrium fluctuations for linear diffusion dynamics. Phys. Rev. E.

[19] Kwon C., Noh J.D., Park H. (2013). Work fluctuations in a time-dependent harmonic potential: Rigorous results beyond the overdamped limit. Phys. Rev. E.

[20] Holubec V., Dierl M., Einax M., Maass P., Chvosta P., Ryabov A. (2015). Asymptotics of work distribution for a Brownian particle in a time-dependent anharmonic potential. Phys. Scr..

[21] Xiao B., Li R. (2019). Work fluctuation and its optimal extraction with time dependent harmonic potential from a non-Markovian bath. Phys. A Stat. Mech. Its Appl..

[22] Paraguassú P.V., Morgado W.A.M. (2021). The heat distribution in a logarithm potential. J. Stat. Mech. Theory Exp..

[23] Pal A., Sabhapandit S. (2014). Work fluctuations for a Brownian particle driven by a correlated external random force. Phys. Rev. E.

[24] Salazar D.S.P. (2020). Work distribution in thermal processes. Phys. Rev. E.

[25] Albay J.A.C., Kwon C., Lai P.Y., Jun Y. (2020). Work relation in instantaneous-equilibrium transition of forward and reverse processes. New J. Phys..

[26] Rosinberg M.L., Tarjus G., Munakata T. (2016). Heat fluctuations for underdamped Langevin dynamics. EPL Europhys. Lett..

[27] Crisanti A., Sarracino A., Zannetti M. (2017). Heat fluctuations of Brownian oscillators in nonstationary processes: Fluctuation theorem and condensation transition. Phys. Rev. E.

[28] Corberi F., Sarracino A. (2018). Probability distributions with singularities. Entropy.

[29] Ciliberto S., Laroche C. (1998). An experimental test of the Gallavotti-Cohen fluctuation theorem. J. Phys. IV.

[30] Garnier N., Ciliberto S. (2005). Nonequilibrium fluctuations in a resistor. Phys. Rev. E.

[31] Douarche F., Joubaud S., Garnier N.B., Petrosyan A., Ciliberto S. (2006). Work fluctuation theorems for harmonic oscillators. Phys. Rev. Lett..

[32] Douarche F., Ciliberto S., Petrosyan A., Rabbiosi I. (2005). An experimental test of the Jarzynski equality in a mechanical experiment. Europhys. Lett..

[33] Reimann P. (2002). Brownian motors: Noisy transport far from equilibrium. Phys. Rep..

[34] Gnoli A., Petri A., Dalton F., Gradenigo G., Pontuale G., Sarracino A., Puglisi A. (2013). Brownian Ratchet in a Thermal Bath Driven by Coulomb Friction. Phys. Rev. Lett..

[35] Gnoli A., Sarracino A., Petri A., Puglisi A. (2013). Non-equilibrium fluctuations in frictional granular motor: Experiments and kinetic theory. Phys. Rev. E..

[36] Di Leonardo R., Angelani L., Dell’Arciprete D., Ruocco G., Iebba V., Schippa S., Conte M.P., Mecarini F., De Angelis F., Di Fabrizio E. (2010). Bacterial ratchet motors. Proc. Natl. Acad. Sci. USA.

[37] Kim H.S., Kim J.H., Kim J. (2011). A review of piezoelectric energy harvesting based on vibration. Int. J. Precis. Eng. Manuf..

[38] Du S., Jia Y., Zhao C., Amaratunga G.A.J., Seshia A.A. (2018). A Passive Design Scheme to Increase the Rectified Power of Piezoelectric Energy Harvesters. IEEE Trans. Ind. Electron..

[39] Costanzo L., Schiavo A.L., Vitelli M. (2020). Active Interface for Piezoelectric Harvesters Based on Multi-Variable Maximum Power Point Tracking. IEEE Trans. Circuits Syst. I Regul. Pap..

[40] Brenes A., Morel A., Juillard J., Lefeuvre E., Badel A. (2020). Maximum power point of piezoelectric energy harvesters: A review of optimality condition for electrical tuning. Smart Mater. Struct..

[41] Costanzo L., Vitelli M. (2020). Tuning Techniques for Piezoelectric and Electromagnetic Vibration Energy Harvesters. Energies.

[42] Halvorsen E. (2008). Energy harvesters driven by broadband random vibrations. J. Microelectromechanical Syst..

[43] Costanzo L., Schiavo A.L., Vitelli M. (2019). Power Extracted From Piezoelectric Harvesters Driven by Non-Sinusoidal Vibrations. IEEE Trans. Circuits Syst. I Regul. Pap..

[44] Quaranta G., Trentadue F., Maruccio C., Marano G.C. (2018). Analysis of piezoelectric energy harvester under modulated and filtered white Gaussian noise. Mech. Sys. Signal Proc..

[45] Cryns J.W., Hatchell B.K., Santiago-Rojas E., Silvers K.L. (2013). Experimental Analysis of a Piezoelectric Energy Harvesting System for Harmonic, Random, and Sine on Random Vibration. Adv. Acoust. Vib..

[46] Risken H. (1989). The Fokker–Planck Equation: Methods of Solution and Applications.

[47] Sekimoto K. (2010). Stochastic Energetics.

[48] van Zon R., Cohen E.G.D. (2003). Extension of the Fluctuation Theorem. Phys. Rev. Lett..

[49] Gammaitoni L., Neri I., Vocca H. (2009). Nonilinear oscillators for vibration energy harvesting. Appl. Phys. Lett..

[50] Cottone F., Vocca H., Gammaitoni L. (2009). Nonlinear Energy Harvesting. Phys. Rev. Lett..

